# Supplementary material for: The discovery of three-dimensional Van Hove singularity
Source: Nat Commun. 2024 Mar 14;15:2313. doi: 10.1038/s41467-024-46626-9 (PMC10940667; doi:10.1038/s41467-024-46626-9)
Supplement: Supplementary file 1 — Supplementary Information [file 41467_2024_46626_MOESM1_ESM.pdf]

Supplementary Information for
The discovery of three-dimensional Van Hove singularity

Wenbin Wu[1,2,3†], Zeping Shi[1†], Mykhaylo Ozerov[4], Yuhan Du[1], Yuxiang Wang[5], Xiao-Sheng Ni[6], Xianghao Meng[1], Xiangyu Jiang[1], Guangyi Wang[1], Congming Hao[1], Xinyi Wang[1], Pengcheng Zhang[1], Chunhui Pan[7], Haifeng Pan[1], Zhenrong Sun[1], Run Yang[8], Yang Xu[2], Yusheng Hou[6], Zhongbo Yan[6], Cheng Zhang[5,9], Hai-Zhou Lu[10], Junhao Chu[2,11], Xiang Yuan[1,2,3*]

[1]State Key Laboratory of Precision Spectroscopy, East China Normal University, Shanghai 200241, China
[2]Key Laboratory of Polar Materials and Devices, Ministry of Education, School of Physics and Electronic Science, East China Normal University, Shanghai 200241, China
[3]Shanghai Center of Brain-Inspired Intelligent Materials and Devices, East China Normal University, Shanghai 200241, China
[4]National High Magnetic Field Laboratory, Florida State University, Tallahassee, Florida 32310, USA
[5]State Key Laboratory of Surface Physics and Institute for Nanoelectronic Devices and Quantum Computing, Fudan University, Shanghai 200433, China
[6]Guangdong Provincial Key Laboratory of Magnetoelectric Physics and Devices, School of Physics, Sun Yat-Sen University, Guangzhou 510275, China
[7]Multifunctional Platform for Innovation Precision Machining Center, East China Normal University, Shanghai 200241, China
[8]Key Laboratory of Quantum Materials and Devices of Ministry of Education, School of Physics, Southeast University, Nanjing 211189, China
[9]Zhangjiang Fudan International Innovation Center, Fudan University, Shanghai 201210, China
[10]Shenzhen Institute for Quantum Science and Engineering and Department of Physics, Southern University of Science and Technology (SUSTech), Shenzhen 518055, China
[11]Institute of Optoelectronics, Fudan University, Shanghai 200438, China

[†]These authors contributed equally to this work.
[*]Correspondence and requests for materials should be addressed to X.Y. (E-mail: xyuan@lps.ecnu.edu.cn)

Table of Content

I. **Characterization of $EuCd_2As_2$ single crystal**

II. **Van Hove singularity in different dimensions**

III. **Energy dispersion around 3D Van Hove singularity and density of states in anisotropic cases**

IV. **Raw spectra and determination of optical transitions**

V. **Satellite peaks and Kerr rotation**

VI. **Band shift with exchange interaction**

VII. **Discussions on local flat bands**

VIII. **Band parameters extracted from the magneto-mid-infrared spectrum**

IX. **Temperature-dependent resistivity**

X. **Optical transitions around the critical field**

XI. **Optical transitions and energy crossing in magneto-near-infrared spectra**

XII. **Density of states calculation**

XIII. **Fermi surface variation and topological Lifshitz transition**

XIV. **Magneto-infrared spectra in Voigt geometry**

I. Characterization of $EuCd_2As_2$ single crystal

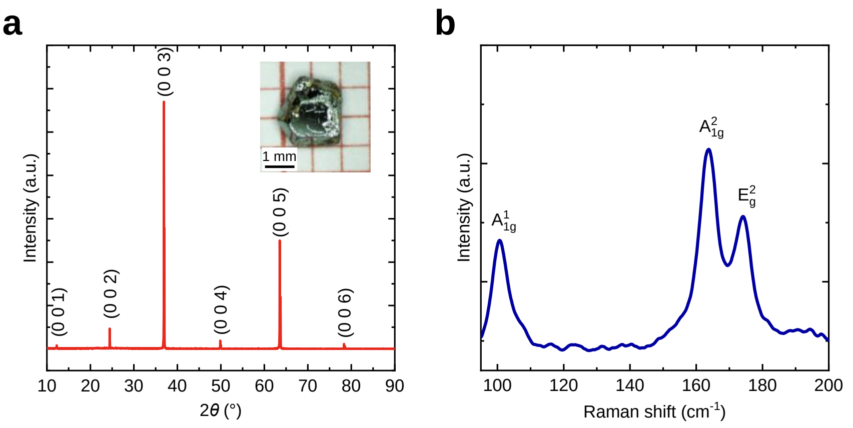

Fig. S1 X-ray diffraction pattern and Raman spectroscopy of $EuCd_2As_2$. a, Single crystal X-ray diffraction pattern along [0 0 1] direction. The inset is the image of the as-grown $EuCd_2As_2$ crystal synthesized by using the Sn-flux method. **b,** Raman spectrum at room temperature using a 632.8 nm He-Ne laser excitation. The A^1_{1g}, A^2_{1g}, E^2_g modes [1] are found at 100.6 cm^{-1}, 163.8 cm^{-1}, 174.2 cm^{-1}, respectively.

II. Van Hove singularity in different dimensions

Van Hove singularity (VHS) is defined [2] as the critical points $\mathbf{k_0}$ at energy band satisfying $|\nabla E(\mathbf{k})|_{\mathbf{k}=\mathbf{k_0}} = 0$, which leads to a divergent peak in the density of states (DOS) at the same time. The Taylor series around the $\mathbf{k_0}$ point reads,

$$E(\mathbf{k}) = E_0 + \Sigma_{i=1}^{d} a_i (k_i - k_{0i})^2, E_0 = E(\mathbf{k_0}) \qquad (1)$$

where d is the dimension, and a_i refers to the second derivative of energy dispersion $\partial^2 E(\mathbf{k})/\partial k_i^2$. According to the sign of a_i, critical points are classified as band extremum (minimum or maximum) and saddle point. The types of critical points in different dimensions and corresponding DOS are summarized in Table S1. One- (1D) and two- (2D) and three-dimensional (3D) critical points are denoted by $Q_{0,1}$, $P_{0,1,2}$ and $M_{0,1,2,3}$, respectively. The subscript is the number of negative a_i. 2D saddle point P_1 and 1D band extrema Q_0, Q_1 serve as VHS revealling divergence in DOS, while all types of 3D critical points exhibit non-divergent DOS, which leads to the absence of 3D VHS.

Table S1 DOS near E_0 in the different dimensions

One-dimensional critical points

Type of critical point	The number of negative a_i	Density of states	
Q_0 Minimum	0	$\mathrm{DOS} \propto 1/\sqrt{E-E_0}\;\; E>E_0$	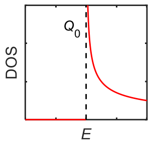
Q_1 Maximum	1	$\mathrm{DOS} \propto 1/\sqrt{E_0-E}\;\; E<E_0$	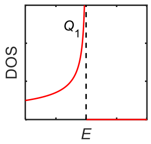

Two-dimensional critical points

Type of critical point	The number of negative a_i	Density of states	
P_0 Minimum	0	$\mathrm{DOS} = \begin{cases} 0 & E<E_0 \\ \mathrm{const} & E>E_0 \end{cases}$	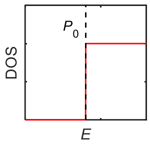
P_1 Saddle point	1	$\mathrm{DOS} \propto -\ln\left\lvert 1-\frac{E}{E_0}\right\rvert$	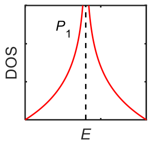
P_2 Maximum	2	$\mathrm{DOS} = \begin{cases} \mathrm{const} & E<E_0 \\ 0 & E>E_0 \end{cases}$	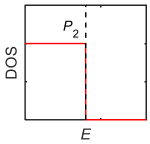

Three-dimensional critical points

Type of critical point	The number of negative a_i	Density of states	
M_0 Minimum	0	$\mathrm{DOS} \propto \sqrt{E-E_0}\;\; E>E_0$	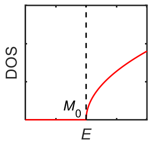
M_1 Saddle point	1	$\mathrm{DOS} \propto \begin{cases} \mathrm{const}-\sqrt{E_0-E} & E<E_0 \\ \mathrm{const} & E>E_0 \end{cases}$	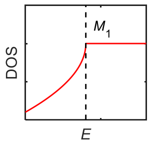
M_2 Saddle point	2	$\mathrm{DOS} \propto \begin{cases} \mathrm{const} & E<E_0 \\ \mathrm{const}-\sqrt{E-E_0} & E>E_0 \end{cases}$	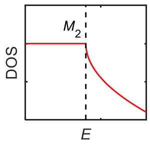
M_3 Maximum	3	$\mathrm{DOS} \propto \sqrt{E_0-E}\;\; E<E_0$	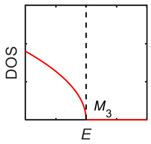

III. Energy dispersion around 3D Van Hove singularity and density of states in anisotropic cases

According to Ref. [3,4], there exist two types of VHS classified by,

$$\nabla_{\mathbf{k}} E = 0 \text{ and } \det D < 0, \tag{2}$$

$$\nabla_{\mathbf{k}} E = 0 \text{ and } \det D = 0. \tag{3}$$

Here, Eq. (2) and (3) describe ordinary and high-order VHS, respectively; $D_{ij} = \frac{1}{2}\partial_i \partial_j E$ is the 2×2 Hessian matrix around VHS $\mathbf{k}_\mathrm{v}$, which can be diagonalized by rotating the axes. The Taylor series of energy dispersion around $\mathbf{k}_\mathrm{v}$ in $\mathbf{p} = \mathbf{k} - \mathbf{k}_\mathrm{v}$ is $E - E_\mathrm{VHS} = -\alpha p_x^2 + \beta p_y^2 + \cdots$. $-\alpha$ and β are eigenvalues of D. When the Eq. (2) are satisfied, the in-plane band structure transforms from a convexity (Fig. S2a) to a Mexican hat (Fig. S2b). With the in-plane isotropy, the band structure is circularly symmetric. The formed critical ring is flat along the tangent direction, as denoted by an orange dotted ring in Fig. S2b, on which each point is equivalent. For simplicity, we focus on the critical point $\mathbf{k}_\mathrm{v}$ located at the k_x axis (pointed out by red dot in Fig. S2b) to analyze the divergence behavior of this critical ring. The energy dispersion along in-plane direction is determined by:

$$E_{xy}(\mathbf{k}_\parallel) = \pm\sqrt{\left(\Delta - mk_x^2 - mk_y^2\right)^2 + v_{xy}^2\left(k_x^2 + k_y^2\right)}. \tag{4}$$

We obtain $\mathbf{k}_{\mathrm{v}\parallel} = (\frac{\sqrt{v_z^2 - 2v_{xy}^2}}{2m}, 0)$ with $v_z = 2\sqrt{\Delta m}$ by solving $\nabla_{\mathbf{k}_\parallel} E_{xy} = 0$. Importantly, only when $v_z \geq \sqrt{2} v_{xy}$, the critical point is well-defined, which is the origin of the critical condition. Owing to the circular symmetry of the in-plane band, we can examine the dispersion around the critical point based on the tangent direction q_tan and radical direction q_rad of the critical ring, as illustrated in Fig. S2b. They are mutually orthogonal and can be obtained by rotating k_x-k_y. The direction perpendicular to the q_tan-q_rad plane is denoted by q_z which is parallel to k_z. Qualitatively, the in-plane energy dispersion around the critical point is flat along q_tan and convex along q_rad. We examine the energy dispersion utilizing Taylor expansion following Ref. [3], and the quadratic terms are given by

$$E_\mathrm{v}(\mathbf{q}) = -E_{\mathrm{v}0} - \alpha q_\mathrm{rad}^2 + \beta q_z^2 + \cdots, \text{with } E_{\mathrm{v}0} = \frac{v_{xy}\sqrt{v_z^2 - v_{xy}^2}}{2m}. \tag{5}$$

Here, the coefficient factors of quadratic terms are $\alpha = \frac{m}{v}\frac{v_z^2 - 2v_{xy}^2}{\sqrt{v_z^2 - v_{xy}^2}}, \beta = \frac{m v_{xy}}{\sqrt{v_z^2 - v_{xy}^2}}$. With $v_z \geq \sqrt{2} v_{xy}$, the condition $\det D_{E_\mathrm{v}} < 0$ is satisfied, indicating that this critical point is an effective 2D saddle point (ordinary VHS) [3], which explains the logarithmically divergent DOS calculated by our band model. The second-order approximation of energy dispersion along q_rad and that along q_z are plotted by blue and yellow dotted curves in Fig. S2c, respectively. It is noteworthy that the prerequisite for accessing this 3D VHS is the formation of a critical ring, rather than the presence of Weyl nodes.

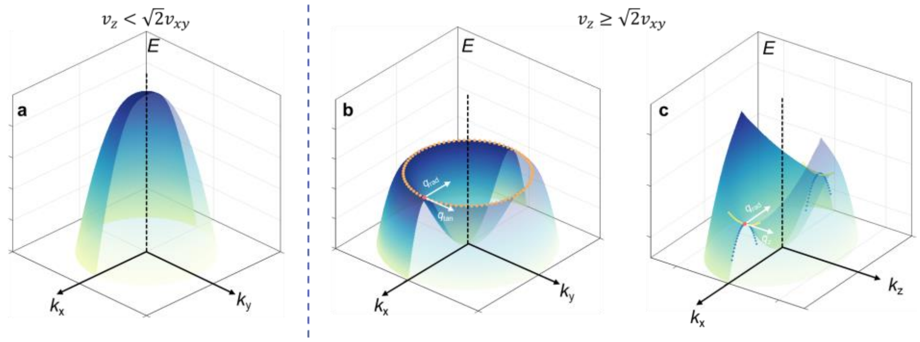

Fig. S2 Comparison between band structure with (without) critical condition satisfied in the isotropic case. a, Energy dispersion along the in-plane direction with $v_z < \sqrt{2}v_{xy}$. **b,** When $v_z \geq \sqrt{2}v_{xy}$, a critical ring appears as denoted by the orange dotted curve. The critical point we discussed is highlighted by a red dot with tangent q_{tan} and radical direction q_{rad} indicated by white arrows. **c,** Energy dispersion in k_x-k_z plane. The red dot represents the same in **b** with radical q_{rad} and out-of-plane direction q_z indicated by white arrows. The second-order approximations of energy dispersion around the critical point along q_{rad} and q_z are plotted in blue and yellow dotted curves, respectively.

When in-plane isotropy is broken, the energy dispersion is described by,

$$E(\mathbf{k}) = \pm\sqrt{\left(\Delta - m_x k_x^2 - m_y k_y^2 - m_z k_z^2\right)^2 + v_x^2 k_x^2 + v_y^2 k_y^2}. \tag{6}$$

The in-plane anisotropy is determined by m_x/m_y and v_x/v_y. The critical condition for formation of saddle ring is given by,

$$v_z \geq v_x\sqrt{\frac{2m_z}{m_x}} \text{ and } v_z \geq v_y\sqrt{\frac{2m_z}{m_y}} \tag{7}$$

As shown in the panel iii in Fig. S3a, when both $v_x/v_z \leq \sqrt{m_x/2m_z}$ and $v_y/v_z \leq \sqrt{m_y/2m_z}$ are satisfied, a loop of saddle points is formed (denoted by the orange dashed ring). To distinguish from the critical ring in the isotropic case, we refer to this loop of saddle points as a saddle ring. Notably, this ring exhibits dispersion along its tangential direction, which differs from the flat ring without dispersion in the isotropic case illustrated in Fig. S2b. In the anisotropic case, the profile of DOS is mainly determined by the saddle points (denoted by white and green dots) located at the extrema of the saddle ring with energies $E_{s1} = v_x\sqrt{4\Delta m_x - v_x^2}/2m_x$ and $E_{s2} = v_y\sqrt{4\Delta m_y - v_y^2}/2m_y$. We individually analyze DOS spectrum with in-plane anisotropy induced by parabolic parameters m_x, m_y and linear parameters v_x, v_y as presented in Fig. S3b. In the isotropic case, the DOS spectrum (black curve in Fig. S3b) exhibits a significant peak with logarithmical divergence which is the origin of our spectroscopic observations. Considering slight anisotropy in the band structure, the

DOS spectrum still shows a sharp peak with $v_x/v_y = 0.95$ or $m_x/m_y = 0.95$ due to the negligible energy difference between E_{s1} and E_{s2}. Like the isotropic case, peak features are expected to be observed in infrared spectrum. With anisotropy extent enhancing, the energy difference between two saddle points increases. Thus, the sharp peak observed in the isotropic case becomes a broad feature with two kinks corresponding to the E_{s1} and E_{s2} similar to DOS spectrum. Due to the band broadening caused by thermal effect or impurity scattering, the DOS spectrum of the real system will be much smoother compared to the calculation shown in Fig. S3b. The spectral profile of the optical transition is generally associated with that of DOS. Therefore, the experimental features might be a broad peak, even drowned in the spectral background, distinct from the sharp features observed in our experiment.

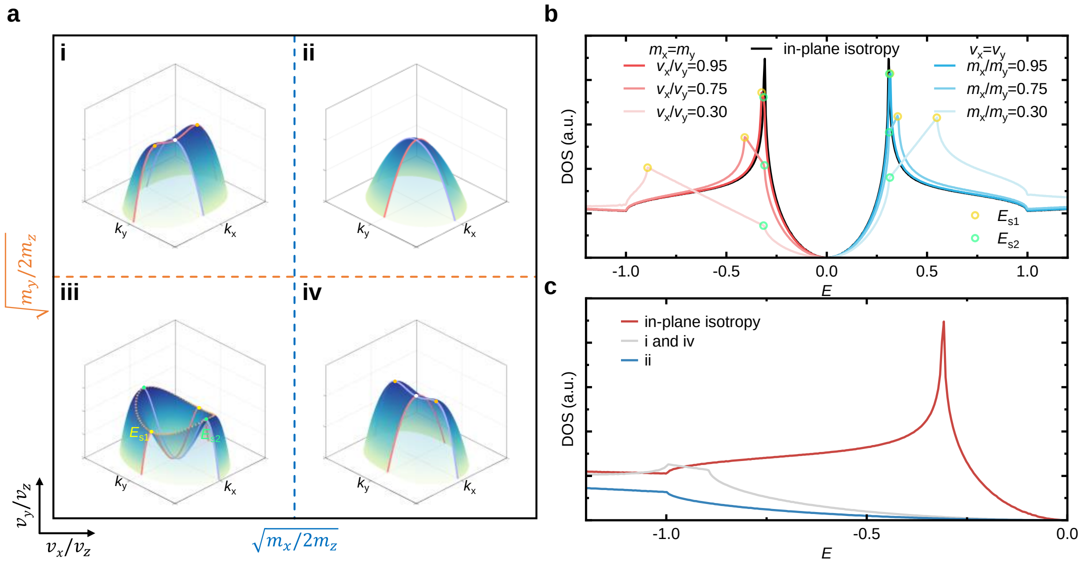

Fig. S3 In-plane energy dispersion with anisotropic in-plane band parameters and corresponding DOS. a, Different cases of band structure with in-plane anisotropy. The energy dispersion along k_x and k_y directions are plotted in red and purple curves. Panels **i** and **iv** show the band structure when energy dispersion along k_x or k_y direction satisfying the critical condition. Two types of critical points, band extremum at zero momentum and saddle points at finite momentum are indicated by white and orange dots, respectively. The band structure in Panel **iii** fully satisfies the critical condition with two types of saddle located at energies E_{s1} and E_{s2} denoted by yellow and green dots. Panel **ii** presents band structure without critical condition satisfied. **b,** DOS spectrum of case **iii** band structure with band anisotropy. The energies of saddle points E_{s1} and E_{s2} are denoted by green and yellow circles, respectively. **c,** Calculated DOS spectrum at different cases. The energy axes in **b** and **c** are normalized by the energy at zero momentum.

Owing to the band anisotropy, as illustrated in the i panel and iv panel of Fig. S3a, two additional cases of band structure exist with a portion of the critical condition Eq. (7)

satisfied. Notably, these two cases are equivalent in essence since k_x and k_y directions can be exchanged. A comparison between DOS spectra of anisotropic and isotropic cases is provided in Fig. S3c. The DOS spectrum (grey curve) possesses two kinks stemming from saddle points located at zero and finite momenta denoted by white and orange points in Fig. S3a, respectively. Compared to the DOS of the isotropic case (red curve in Fig. S3c), the DOS of cases i and iv are much lower and broader, which might be hard to contribute to prominent peak features in spectroscopy. As for the band structure in panel ii of Fig. S3a, the critical condition is not satisfied. Therefore, regardless of whether the band is isotropic, the DOS of this case (blue curve in Fig. S3c) shows a finite kink induced by the 3D saddle at zero momentum that is fully consistent with the experimental features below the critical field.

IV. Raw spectra and determination of optical transitions

As the NHMFL data shown in Fig. S4a, the peak features of T_{α} and T_{β} are pointed out by blue and red arrows, respectively. The optical transitions in the raw data exhibit a "3-stage" variation: being low but observable below B_{c}; increasing sharply after reaching B_{c}; becoming saturated after the magnetic saturation field of magnetization B_{s}. The signal-to-noise level of NHMFL spectra is comparatively lower owing to the much lower detectivity of the bolometer in the high-energy regime. That leads to lower intensity and broader linewidth of spectral features compared to ECNU spectra (Fig. S4b). Therefore, these optical features are better resolved in the false-color plot (Fig. 2c in the main text), where broad features are usually better visualized.

As for the ECNU data illustrated in Fig. S4b, T_{β} and its satellite peaks can be well determined because of the much lower noise level compared to the NHMFL data. The T_{α} overlaps with satellite peaks at high fields. Satellite peaks dominate the spectrum due to much lower narrower widths and larger amount of features. Once T_{α} overlaps with satellite peaks, it will be more challenging to be directly visualized but still can be resolved in the stacking plot (Fig. S4b). Once again, such broad features with overlapping conditions are naturally better resolved in the false-color plot as shown in Fig. 2d of the main text. Thus, we conduct the Drude-Lorentz function [5] to analyze the ECNU data. The dielectric function Drude-Lorentz model reads,

$$\epsilon(\omega) = \epsilon_{\infty} - \sum_i \frac{\omega_{p,D;i}^2}{\omega^2 + \frac{i\omega}{\tau_{D,i}}} + \sum_j \frac{\Omega_j^2}{\omega_j^2 - \omega^2 - i\omega\gamma_j}, \tag{8}$$

where ω is the frequency; ϵ_{∞} is the real part at high frequency; the first sum denotes free carrier response with plasma frequency $\omega_{p,D;i}$ and scattering rate $1/\tau_{D,i}$ of the ith Drude component; the second sum denotes the bound excitations with strength Ω_j, energy position ω_j and linewidth γ_j of the jth Lorentz component. To fit with the experimental spectrum, we calculate the reflectivity utilizing Eq. (8) without the Drude

term (the first sum). The fitting result at 7 T (orange curve in Fig. S4c) agrees well with the corresponding experimental spectrum (blue curve in Fig. S4c), which indicates the energies of the observed transition are very close to apparent peak features in magneto-reflectivity spectra in Fig. S4b. Therefore, it is reasonable to extract the transition energy by the apparent peak position. Combining with scaling parameters (linear relation between the peak energy and magnetization) extracted from experiments and DOS calculated by the two-band model, we successfully reproduce the entire spectra utilizing the Drude-Lorentz function as shown in the right panel of Fig. S4d, which is consistent with the experiment spectra shown in the left panel.

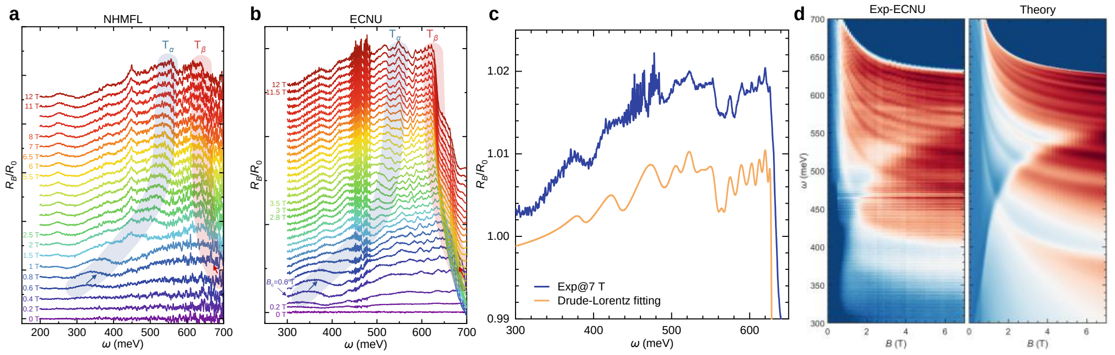

Fig. S4 Magneto-infrared spectra and determination of optical transitions. a, NHMFL data up to 12 T. The peak features of T_α and T_β are pointed out by blue and red arrows, respectively. **b,** ECNU data up to 12 T. For clarification, the constant offsets are introduced in stacking plots of spectra. **c,** Experimental spectrum at 7 T and Drude-Lorentz fitting result. A constant offset is introduced for clarification. **d,** The left and right panels are the false-color plots of ECNU data and spectra reproduced by the Drude-Lorentz function, respectively.

V. Satellite peaks and Kerr rotation

As shown in Fig. S5a, the satellite peak series can be divided into two groups according to relatively strong and weak intensity (hereafter referred to as ‘strong peak series’ and ‘weak peak series’). The stacking plot of the spectra is shown in Fig. S4b where the satellite peaks can be visualized. The energies of all strong and weak peaks are extracted in Fig. S5b, as denoted by hollow squares and solid diamonds, respectively. The separation between adjacent peak series in each series becomes narrower in higher energy. Above ~ 600 meV, the separation between the strong peak series is too narrow to distinguish the weak peak series. The energy variation of each peak series is consistent with the linear scaling (solid curves with corresponding light color in Fig. S5b) of 8 K magnetization exhibited in Fig. S5c.

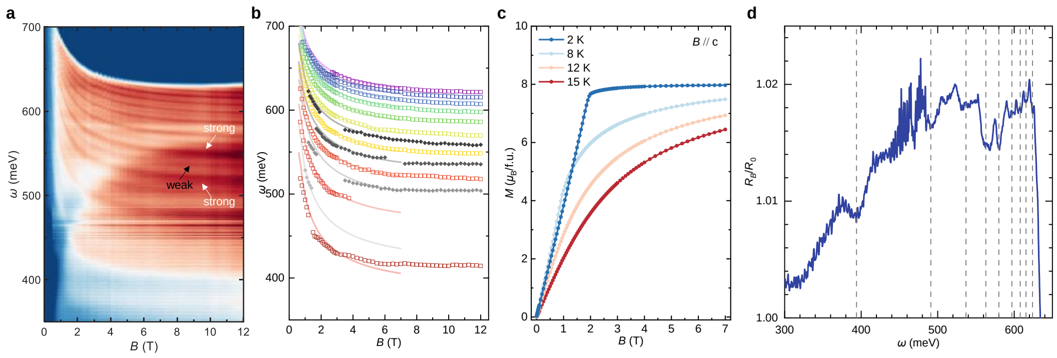

Fig. S5 The satellite peaks in mid-infrared spectroscopy. a, False-color plot of magneto-infrared spectra in mid-infrared regime. The satellite peaks are distinguished and categorized into two series based on their intensity. **b,** The extracted energies of satellite peaks are denoted by hollow squares (for the strong series) and solid diamonds (for the weak series). The energy variation of satellite peaks with fields is consistent with the linear scaling of the magnetization at 8 K. The linear scaling curves are presented in lighter colors of the corresponding series of satellite peaks. **c,** The magnetization at different temperatures. **d,** Magneto-reflectivity spectrum measured at 7 T in ECNU. A series of satellite peaks can be visualized. The separation between adjacent minima decreases at higher energy. The satellite features merge and terminate around 630 meV.

For the Kerr/Faraday rotation [6–8] of the magnetic materials and spin-polarized systems, the smoking-gun phenomenon is a series of oscillating satellite structures with the energy period decreasing at higher energy and terminating at the highest-energy feature related to the corresponding interband transition. As shown in Fig. S5d, the local minima are indicated by dashed grey lines. The spacing between adjacent minima becomes narrower at higher energy, which agrees with previous reports. Under external magnetic fields, electronic bands of $EuCd_2As_2$ become spin-polarized due to exchange interaction. Therefore, this system exhibits different reflectivity for left-handed (LCP) and right-handed circularly polarized (RCP) lights. The difference between LCP and RCP reflectivity depends periodically on wavelengths and depends linearly on the magnetization. Therefore, the combined reflectivity exhibits an oscillating satellite structure. Importantly, the energy dispersion of satellite peaks as well as their energy separation are expected to follow the linear relation on the magnetization instead of magnetic fields. This phenomenon could be amplified if two linear polarizers parallel (perpendicular) to each other are mounted in the incident and exit light paths [8]. The consistency of the presence of satellite structures and decreasing period at higher energy indicates the Kerr rotation as the possible origin of the observed satellite peaks. Therefore, T_β with the highest energy in our experiment where the satellite peaks merge and terminate is assigned as the main peak. It also explains the absence of satellite peaks

alongside the transition T_α since the initial state of T_α splits negligibly.

VI. Band shift with exchange interaction

The energy shift of Weyl bands in $EuCd_2As_2$ originates from the spin-canting-induced exchange interaction under the external magnetic field. The exchange interaction [9] between the itinerant electrons and local magnetic moments is described by $H_{\mathrm{exc}} = -J_0 \Sigma \mathbf{s} \cdot \mathbf{S}$, where **s** and **S** is the spin of the itinerant electrons and neighboring Eu^{2+} ions, respectively, with orbital-dependent exchange constant J_0. Under the mean-field approximation, the mean spin of Eu^{2+} surrounding the itinerant electron is given by $\langle \mathbf{S} \rangle = S \cdot M(T,B)/M_{\mathrm{s}}$, which is associated with the magnetization of the system. Here, $M(T,B)$ is the magnetization with the specific temperature and external magnetic field, and M_{s} is the saturation magnetization with Eu^{2+} spin $S = 7/2$. The discussed Weyl bands are from different orbitals, and the Hamiltonian proposed in the main text is rewritten as,

$$H = \begin{pmatrix} \Delta - m\mathbf{k}^2 & v_{xy}(k_x - ik_y) \\ v_{xy}(k_x + ik_y) & -\Delta + m\mathbf{k}^2 \end{pmatrix} + \begin{pmatrix} \frac{1}{2} J_1 S \frac{M}{M_{\mathrm{s}}} & 0 \\ 0 & -\frac{1}{2} J_2 S \frac{M}{M_{\mathrm{s}}} \end{pmatrix}, \quad (9)$$

where J_1 and J_2 are the exchange constants for the electrons from different orbitals. With $\bar{J} = (J_1 + J_2)/2$ and $\delta J = (J_1 - J_2)/2$, the Hamiltonian reads,

$$H = (\Delta - m\mathbf{k}^2)\sigma_z + v_{xy}(k_x\sigma_x + k_y\sigma_y) + \frac{1}{2}\bar{J}S\frac{M}{M_{\mathrm{s}}}\sigma_z + \frac{1}{2}\delta J S \frac{M}{M_{\mathrm{s}}}\sigma_0. \quad (10)$$

The energy dispersion is given by,

$$E = \frac{1}{2}\delta J S \frac{M}{M_{\mathrm{s}}} \pm \sqrt{\left(\Delta + \frac{1}{2}\bar{J}S\frac{M}{M_{\mathrm{s}}} - m\mathbf{k}^2\right)^2 + v_{xy}^2(k_x^2 + k_y^2)}. \quad (11)$$

With the exchange interaction, the Fermi velocity $v_z(M) = 2\sqrt{(\Delta + \frac{1}{2}\bar{J}S\frac{M}{M_{\mathrm{s}}}) \cdot m}$ is effectively controllable by magnetization. From the critical Fermi velocity $v_z = \sqrt{2}v_{xy}$, the critical magnetization is obtained by $M_{\mathrm{c}} = \frac{M_{\mathrm{s}}}{\bar{J}S}(\frac{v_{xy}^2}{m} - 2\Delta)$. The critical B_{c} is the corresponding magnetic field of $M = M_{\mathrm{c}}$.

VII. Discussions on local flat bands

The energy of T_α and that of T_β are beyond the general energy range of the Weyl nodes. Hence, additional electronic bands should be included to explain all observed optical transitions. Suppose that only one upper (UFB) or lower flat band (LFB) contributes to the optical response. This band configuration suggests two optical transitions with identical zero-field extrapolation, which deviates from the observation in the magneto-infrared spectrum. Another critical issue of this band configuration is that the higher-energy optical transition should increase in energy with the magnetic field, and the

lower-energy one is on the contrary. This issue also contrasts with the experimental observation that the lower-energy optical transitions increase in energy with the magnetic field and vice versa. By considering both the UFB and the LFB, one can explain not only the opposite energy evolution of T_α and T_β with magnetic fields but quite different zero-field extrapolation energies of them. The optical transitions between the UFB and the LFB are further observed in the near-infrared spectrum and agree with the proposed band configuration.

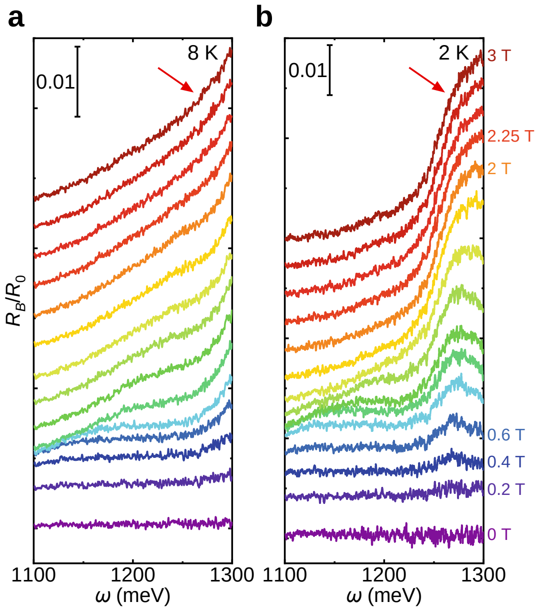

Fig. S6 Magneto-infrared spectroscopy in NIR regime. a, b, magneto-reflectivity R_B/R_0 measured at 8 K and 2 K. The noteworthy peak around 1300 meV (pointed by red arrow) may be the signature of the optical transition associated with Eu 4f levels.

As revealed by the ARPES result and density functional theory (DFT) calculation [10–12], multiple flat bands (from the Eu 4f electrons) stay deeper than 1eV below Fermi energy in $EuCd_2As_2$. But we exclude these flat 4f bands as the origin of observed optical transitions (T_α and T_β) because of the discrepancy in the energy and linewidth. On the aspect of transition energy, 4f bands are located at extremely deep binding energy 1~1.75 eV as demonstrated in previous angle-resolved photoemission spectroscopy (ARPES) results [10]. The corresponding optical transitions are expected to be located at the high energy regime of the spectrum (toward the visible regime). The copper optical pipes and gold-coated focusing mirrors installed in our magneto-infrared setup render the spectral range far below the energy of most related optical transitions. In fact, a pronounced spectral feature is found around 1300 meV as highlighted by red arrows in Fig. S6 which might serve as the possible signature of the optical transition originating from 4f bands. On the aspect of linewidth, 4f bands exhibit significant energy broadening on the energy of 1 eV. It contradicts the small width of observed

optical transitions. Therefore, 4f bands are excluded as the possible origin. For the same reason, the satellite peaks alongside the T_β are confirmed not from the multiple 4f bands. Recent work evidence a semiconductor band structure [13] utilizing time-resolved ARPES and magneto-infrared transmission spectroscopy. The band edge of the conduction band probably plays the role of UFB.

VIII. Band parameters extracted from the magneto-mid-infrared spectrum

The fitted band parameters are summarized in Table S2. Here, Δ, m, v_{xy} are the band parameters for the Weyl bands in the zero magnetic field; J_1 and J_2 are exchange constants of the Weyl bands from the different orbitals; E_{LFB} and E_{UFB} are the zero-field energy of the LFB and the UFB, respectively. The experimental results reveal finite exchange constant J_{UFB} of the UFB but nearly zero exchange splitting of the LFB ($J_{\mathrm{LFB}} \approx 0$).

Table S2 Band parameters

Δ (meV)	m (meV · nm^2)	v_{xy} (meV · nm)	J_1 (meV)	J_2 (meV)	E_{UFB} (meV)	J_{UFB} (meV)	E_{LFB} (meV)
25.5	200.1	202.4	195.1	58.9	759.9	107.9	-249.1

IX. Temperature-dependent resistivity

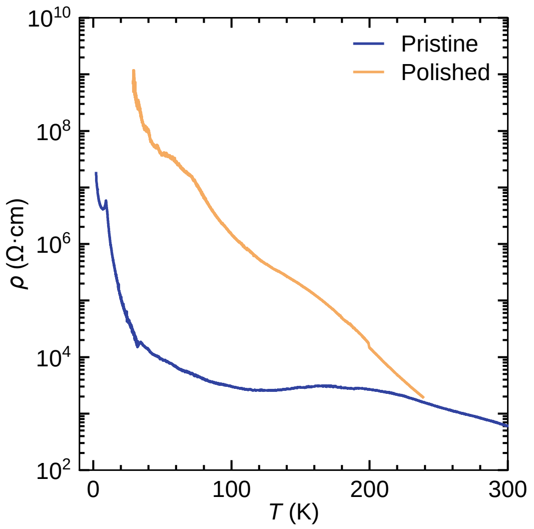

Fig. S7 Temperature-dependent resistivity of our sample measured before and after surface polish. Our sample exhibits insulating behavior in resistivity similar to reported insulating samples [13]. Compared to the pristine sample, the resistivity increases several orders of magnitude in the polished one. It indicates possible unconventional surface states in the sample.

X. Optical transitions around the critical field

Figs. S8a and b exhibit raw spectra measured in ECNU (using our home-built magneto-

infrared setup) and NHMFL, respectively. The peak features of T_α transition are traced to low fields with pink triangles. In both ECNU and NHMFL data, the peak feature of T_α is very weak below the critical field of 0.6 T. An abrupt height increase of T_α peak can be found around 0.6 T, which is related to the formation of the 3D VHS as described by our model. This intensity evolution is better captured in the false-color plots at low field range, as the ECNU and NHMFL spectra shown in Fig. 3a of the main text and Fig. S8c, respectively.

The NHMFL spectra present higher quality below 300 meV (due to the bolometer detector and lower cutoff frequency in the NHMFL setup) and prove the absence of optical features with field-dependent energy. Since the spectra above 300 meV are enough to show the complete field evolution of the optical transitions according to the raw data, we set it as the lowest energy of the false-color plots. In much higher magnetic fields than 0.6 T, T_α can not be directly traced in the stacking plot due to the energy overlap with satellite peaks. The satellite peaks dominate the spectrum behavior due to much narrower linewidth. It also explains why the T_α can be better resolved in the false-color plot. For the same reason, T_α can be better resolved in the stacking plot with the worse signal-to-noise ratio (mid-infrared regime in NHMFL).

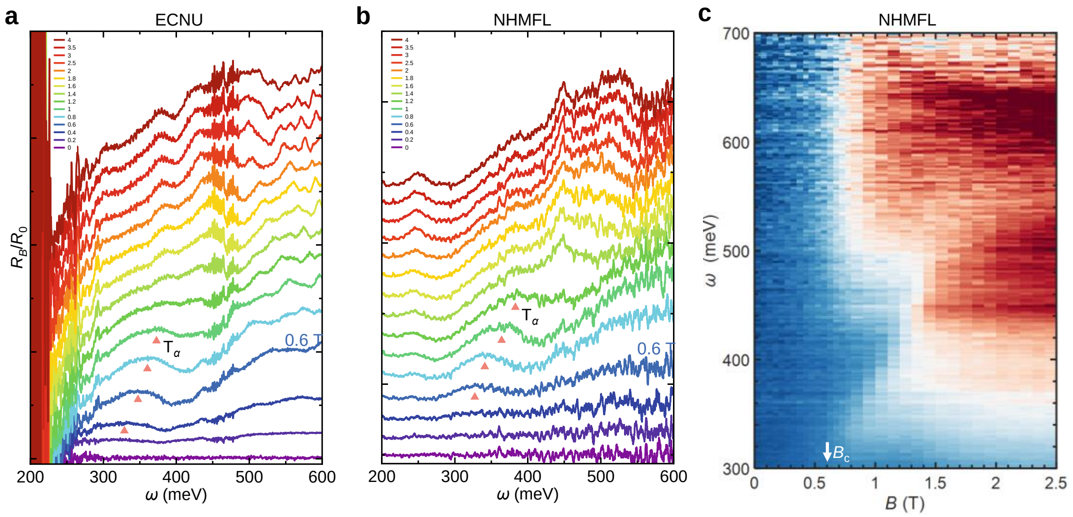

Fig. S8 Low-field magneto-infrared spectra. a, b Stacking plots of magneto-infrared spectra measured in ECNU and NHMFL with peak features of T_α denoted by pink triangle. **c,** False-color plot of NHMFL spectra with the critical field pointed out by the white arrow.

As shown in Fig. S9a, the feature of T_β can be traced below the critical field, which is well predicted theoretically. Based on the proposed model, the overall DOS is contributed by both critical points in zero momentum (non-divergent) and finite momentum (VHS). The energy of these two types of critical points merge at B_c. The

former critical point presents a prominent kink in the DOS, leading to observable T_β below B_c. After reaching B_c, the VHS forms and induced DOS are expected to increase much more sharply than before B_c due to the divergent nature of VHS. The sharp increase of the peak eventually terminates around the magnetic saturation field B_s. Therefore, the model predicts a "3-stage" intensity variation: being low but observable below B_c; increasing sharply after reaching B_c; becoming saturated after the saturation field of the magnetization B_s.

We further try to analyze more quantitively between the experiments and theory, starting from the satellite peaks due to their well-defined peak dip features. The spectral weight of T_β is largely assigned to the satellite peaks. The field dependence is almost identical between T_β and its satellite peak. The following analysis is preliminary because it may take additional caution while dealing with peak height in the magneto-reflectivity spectra.

We choose several well-defined satellite peaks (indicated by arrows in Fig. S9a) and extract the apparent peak positions and heights to track the intensity variation of field-induced optical transitions. As shown in Fig. S9b, peak heights of all satellite peaks follow "3-stage" behavior and prominently rise around the critical field. Before B_c, the peak heights are finite and increase gradually. To compare with the proposed model, we extract the peak height of the predicted DOS in Fig. S9c. The upper panel of Fig. S9c shows the DOS variations with and without energy broadening (red and blue curves) caused by thermal effect or disorder scattering. Broadening energy is set as 0.5 meV. With or without the effect of broadening, the calculated DOS reproduces the "3-stage" behavior and agrees well with the experimental observation.

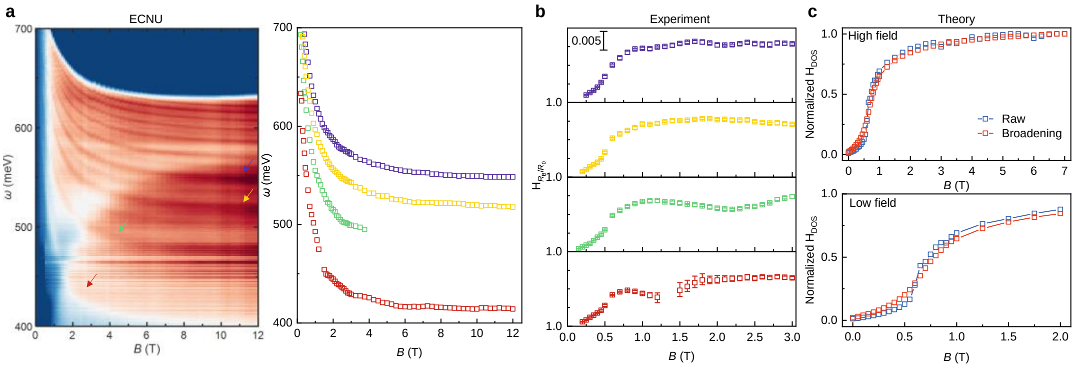

Fig. S9 Comparison between apparent peak height of transition features and that of DOS peak. a, False-color plot of magneto-infrared spectra measured at ECNU. To illustrate the spectral variation around the critical field, we select four series of peaks (denoted by arrows in the left panel) with their energies presented in the right panel. **b,** Extracted apparent peak height H_{R_B/R_0} of selected four series of peaks from magneto-reflectively spectra R_B/R_0 . **c,** Apparent peak height of calculated DOS $H_{\rm DOS}$

normalized by the maximum value. The calculation with (without) energy broadening is denoted by the red (blue) curve. Zoom-in presentation within 2 T is shown in the bottom panel.

XI. Optical transitions and energy crossing in magneto-near-infrared spectra

While Ref. [13] reports the magneto-infrared spectrum of this compound, we focus on the evolution of optical transitions upon magnetization and find additional optical transitions utilizing magneto-near-infrared spectroscopy. As shown in Figs. S10a and b, the peak features of $T_\delta, T_\varepsilon, T_\gamma$ are traced by purple, green and blue triangles, respectively. An obvious energy cross of T_δ and T_γ is found around 0.8 T which is pointed out by white arrows in false-color plots (Figs. S10c and d). These peak features and cross are more clear at 2 K spectra, due to the sharper magnetization variation and lower thermal broadening effect.

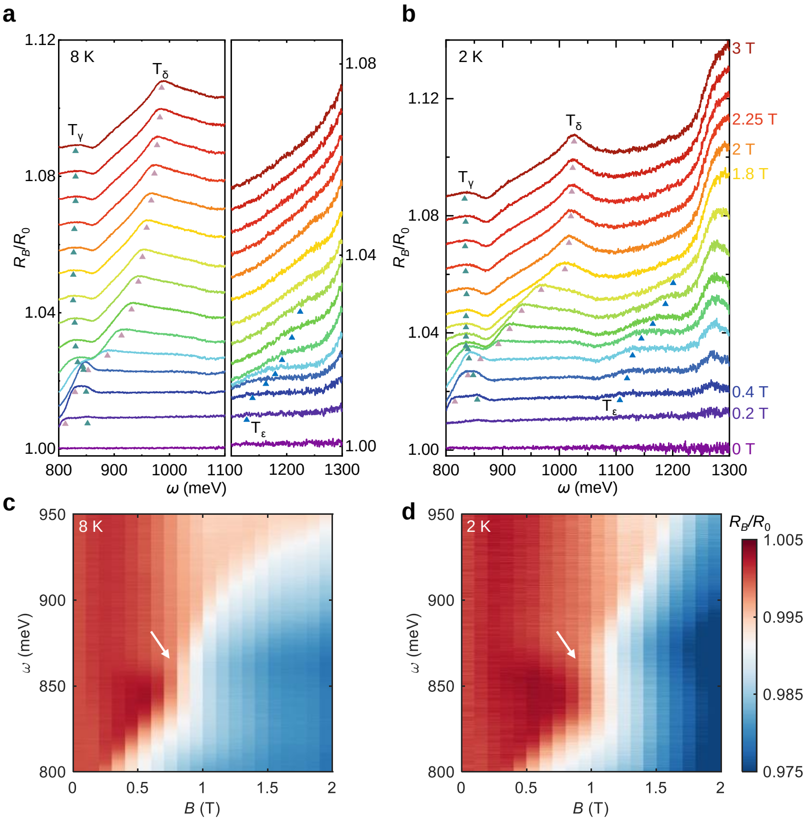

Fig. S10 Zoom-in magneto-near-infrared spectra measured at 8 K and 2K. a-b, Stacking plot of spectra measured at 8 K and 2 K up to 3 T with $T_\delta, T_\gamma, T_\varepsilon$ traced by purple, green and blue triangles, respectively. The left and right panels of **a** adopt different offsets to make all peak features clear. With the field increasing, T_ε merges into prominent peak features that may be attributed to the 4f-bands-related optical transitions. **c-d,** False-color plots of spectra measured at 8 K and 2 K up to 2 T. The energy crossing of optical transitions T_γ and T_δ is pointed by the white arrows.

XII. Density of states calculation

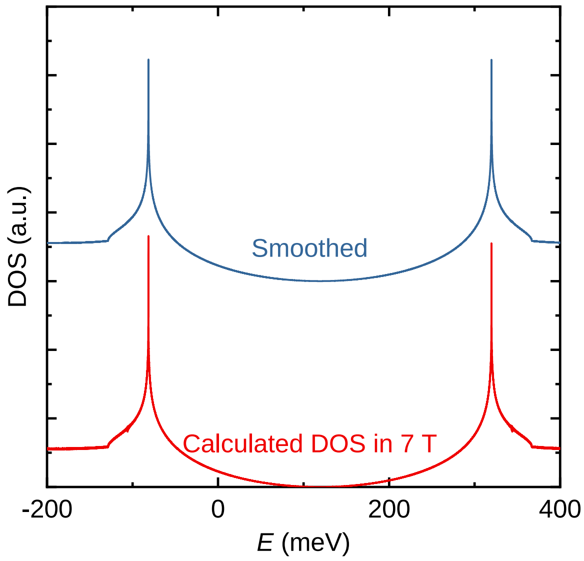

Fig. S11 Raw and smoothed theoretical DOS spectrum in 7 T. The red and blue curves denote the calculated DOS with and without smoothing. Some artificial random spikes appear in the raw theoretical DOS spectrum but are no longer resolved after adjacent-average smoothing with a 5-point span.

XIII. Fermi surface variation and topological Lifshitz transition

Although the observed 3D VHS is not topologically protected, it is connected to the topology of this system. For example, the 3D VHS plays the role of topological Lifshitz transition point when the critical condition is satisfied. For simplicity, we refer to the band structures with/without 3D VHS as "ordinary/VHS case" as illustrated in Figs. S12a and e. We examine the Fermi surface at three characteristic energies E_{F1}, E_{F2}, E_{F3}. At E_{F1}, both cases are in the Weyl semimetal phase with two opposite chiral Weyl pockets as shown in Figs. S12b and f. The topological Lifshitz transition energy E_{F2} corresponds to the zero-momentum energy in ordinary case. In comparison, the 3D VHS serve as the topological Lifshitz transition point in the VHS case which divides the Weyl semimetal phase and trivial metal phase. As presented in Figs. S12c and g, two Weyl pockets merge into a single one, letting the net Berry flux on the Fermi surface vanish. Additional Lifshitz transition is expected in the VHS case when Fermi energy is located below E_{F3} (energy of zero-momentum critical point) as the Fermi surface shown in Figs. S12h, while the topology of Fermi surface remains when Fermi energy drops to E_{F3} in ordinary case (Fig. S12d). Therefore, although 3D VHS is not topologically protected, it is tightly associated with the band topology of $EuCd_2As_2$.

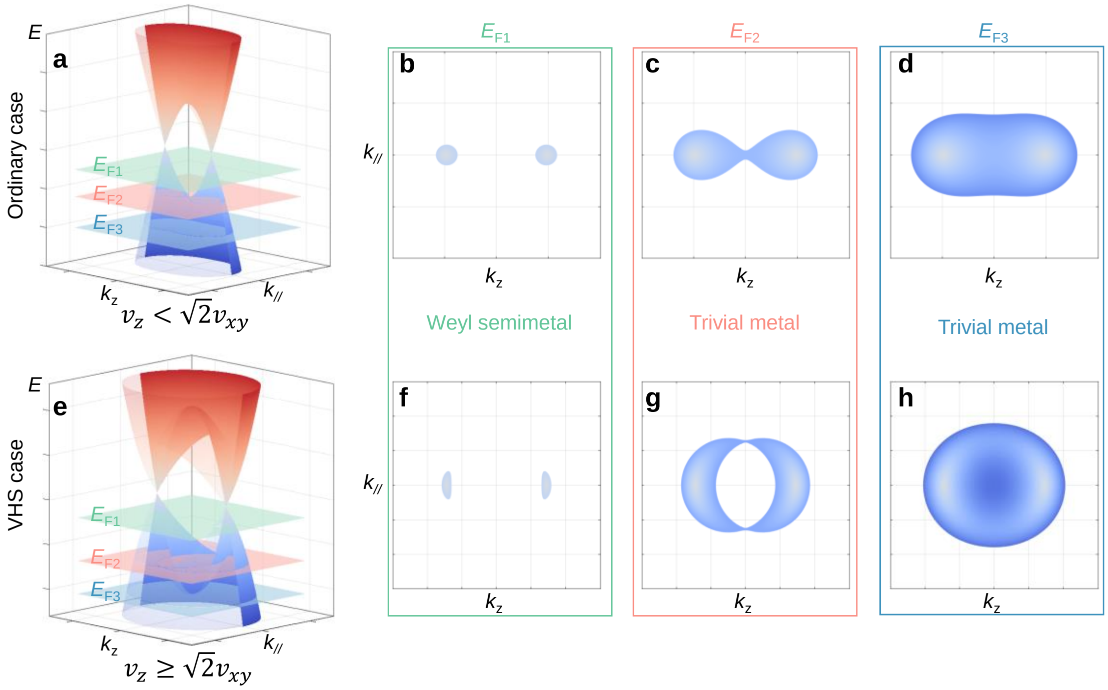

Fig. S12 Fermi surface of Weyl bands with (without) critical condition satisfied. a, e, Energy dispersion of Weyl bands without (with) critical condition satisfied (corresponding to "ordinary (VHS) case"). The examined Fermi energies E_{F1}, E_{F2}, E_{F3} are denoted by the green, red and blue planes, respectively. The Weyl semimetal phase vanishes at E_{F2} where system undergoes a topological Lifshitz transition. **b-d,** Fermi surfaces of Weyl bands with $v_z < \sqrt{2}v_{xy}$ at different energies. In this case, E_{F2} corresponds to the energy of the zero-momentum critical point. **f-h,** Fermi surfaces of Weyl bands with $v_z \geq \sqrt{2}v_{xy}$ at different energies. In this case, E_{F2} corresponds to the energy of 3D VHS. Additionally, an extra Lifshitz transition takes place at E_{F3} corresponding to the energy of the zero-momentum critical point.

XIV. Magneto-infrared spectra in Voigt geometry

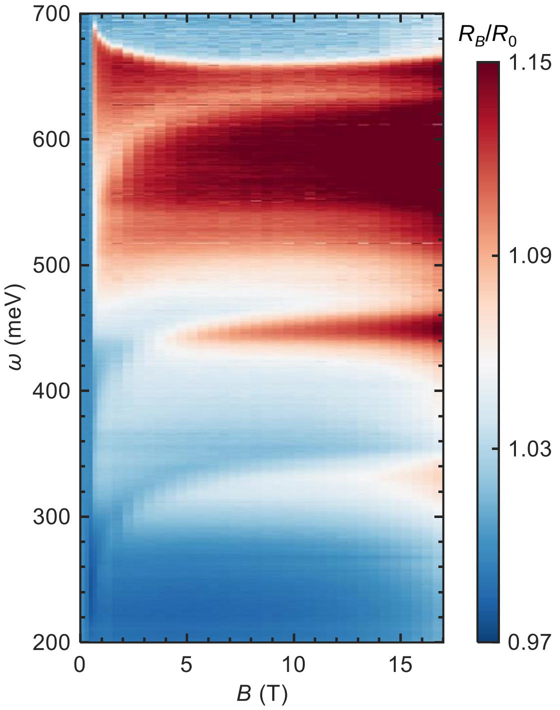

Fig. S13 False-color plot of magneto-infrared spectra in Voigt geometry. The external magnetic field is applied along the in-plane direction (perpendicular to the wave vector of the infrared beam). The abrupt emergence of optical transitions is found at the field which is much lower than the critical field in Faraday geometry.

References

[1] Y. Du, J. Chen, W. Wu, Z. Shi, X. Meng, C. Zhang, S. Gong, J. Chu, and X. Yuan, *Comparative Raman Spectroscopy of Magnetic Topological Material $EuCd_2X_2$ (X=P, As)*, J. Phys.: Condens. Matter **34**, 224001 (2022).

[2] L. Van Hove, *The Occurrence of Singularities in the Elastic Frequency Distribution of a Crystal*, Phys. Rev. **89**, 1189 (1953).

[3] N. F. Q. Yuan, H. Isobe, and L. Fu, *Magic of High-Order van Hove Singularity*, Nat Commun **10**, 5769 (2019).

[4] N. F. Q. Yuan and L. Fu, *Classification of Critical Points in Energy Bands Based on Topology, Scaling, and Symmetry*, Phys. Rev. B **101**, 125120 (2020).

[5] M. Dressel and G. Grüner, *Electrodynamics of Solids: Optical Properties of Electrons in Matter* (Cambridge University Press, 2002).

[6] H. Krenn, W. Herbst, H. Pascher, Y. Ueta, G. Springholz, and G. Bauer, *Interband Faraday and Kerr Rotation and Magnetization of $Pb_{1-x}Eu_xTe$ in the Concentration Range $0<x\leq 1$*, Phys. Rev. B **60**, 8117 (1999).

[7] D. U. Bartholomew, J. K. Furdyna, and A. K. Ramdas, *Interband Faraday Rotation in Diluted Magnetic Semiconductors: $Zn_{1-x}Mn_xTe$ and $Cd_{1-x}Mn_xTe$*, Phys. Rev. B **34**,

6943 (1986).

[8] L. Ohnoutek, M. Hakl, M. Veis, B. A. Piot, C. Faugeras, G. Martinez, M. V. Yakushev, R. W. Martin, Č. Drašar, A. Materna, G. Strzelecka, A. Hruban, M. Potemski, and M. Orlita, *Strong Interband Faraday Rotation in 3D Topological Insulator* Bi_2Se_3, Sci. Rep. **6**, 19087 (2016).

[9] J. S. Moodera, T. S. Santos, and T. Nagahama, *The Phenomena of Spin-Filter Tunnelling*, J. Phys.: Condens. Matter **19**, 165202 (2007).

[10] J.-Z. Ma, S. M. Nie, C. J. Yi, J. Jandke, T. Shang, M. Y. Yao, M. Naamneh, L. Q. Yan, Y. Sun, A. Chikina, V. N. Strocov, M. Medarde, M. Song, Y.-M. Xiong, G. Xu, W. Wulfhekel, J. Mesot, M. Reticcioli, C. Franchini, C. Mudry, M. Müller, Y. G. Shi, T. Qian, H. Ding, and M. Shi, *Spin Fluctuation Induced Weyl Semimetal State in the Paramagnetic Phase of* $EuCd_2As_2$, Sci. Adv. **5**, eaaw4718 (2019).

[11] G. Hua, S. Nie, Z. Song, R. Yu, G. Xu, and K. Yao, *Dirac Semimetal in Type-IV Magnetic Space Groups*, Phys. Rev. B **98**, 201116 (2018).

[12] J.-R. Soh, F. de Juan, M. G. Vergniory, N. B. M. Schröter, M. C. Rahn, D. Y. Yan, J. Jiang, M. Bristow, P. A. Reiss, J. N. Blandy, Y. F. Guo, Y. G. Shi, T. K. Kim, A. McCollam, S. H. Simon, Y. Chen, A. I. Coldea, and A. T. Boothroyd, *Ideal Weyl Semimetal Induced by Magnetic Exchange*, Phys. Rev. B **100**, 201102 (2019).

[13] D. Santos-Cottin, I. Mohelský, J. Wyzula, F. Le Mardelé, I. Kapon, S. Nasrallah, N. Barišić, I. Živković, J. R. Soh, F. Guo, K. Rigaux, M. Puppin, J. H. Dil, B. Gudac, Z. Rukelj, M. Novak, A. B. Kuzmenko, C. C. Homes, T. Dietl, M. Orlita, and A. Akrap, $EuCd_2As_2$*: A Magnetic Semiconductor*, Phys. Rev. Lett. **131**, 186704 (2023).
